# Video head impulse testing before and after canalith repositioning for benign paroxysmal positional vertigo

**DOI:** 10.1007/s00405-026-10263-3

**Published:** 2026-05-05

**Authors:** Thanatporn Vongviriyangkoon, Siriporn Limviriyakul, Kanthong Thongyai, Suvajana Atipas, Sarun Prakairungthong, Kanokrat Suvarnsit

**Affiliations:** Department of Otorhinolaryngology, Faculty of Medicine Siriraj Hospital, Mahidol University, 2 Wanglang Road, Bangkok Noi, Bangkok, 10700 Thailand

**Keywords:** Benign paroxysmal positional vertigo, BPPV, Vestibulo-ocular reflex gain, Video head impulse test

## Abstract

**Objective:**

To evaluate video head impulse testing (vHIT) outcomes in patients with benign paroxysmal positional vertigo (BPPV) before canalith repositioning maneuver (CRM), immediately after treatment, and 1 week post-treatment.

**Study design:**

Prospective before–after study.

**Setting:**

Tertiary referral center.

**Patients:**

Of 156 patients with vertigo and positional nystagmus, 63 were diagnosed with BPPV using standard maneuvers; 51 had posterior canal BPPV, 11 had lateral canal BPPV and 1 had anterior canal BPPV. All underwent vHIT to assess vestibulo-ocular reflex (VOR) gain across all 6 semicircular canals and completed the Dizziness Handicap Inventory before and after treatment.

**Interventions:**

CRMs specific to the affected canal.

**Main outcome measures:**

VOR gain and Dizziness Handicap Inventory scores before and after CRM.

**Results:**

At pre-treatment, 59 patients (93.7%) exhibited VOR gains within normative limits. Only four patients (6.3%) showed decreased VOR gains, all of whom were diagnosed with posterior canal BPPV. VOR gain did not differ significantly across pre-treatment, immediate post-treatment, and 1-week assessments. No corrective saccades were detected.

**Conclusions:**

vHIT showed no significant post-treatment changes and may have limited incremental value in the routine evaluation of isolated BPPV cases.

**Supplementary Information:**

The online version contains supplementary material available at 10.1007/s00405-026-10263-3.

## Introduction

Benign paroxysmal positional vertigo (BPPV) is a common peripheral vestibular disorder, accounting for 17%–40% of cases and occurring more often in females [1–3]. Brief, position-triggered vertigo occurs when displaced otoconia enter the semicircular canals [1, 4]. The posterior canal is affected in 85%–95% of cases, while the lateral and anterior canals are involved less frequently [1, 4]. Diagnosis integrates history with observation of nystagmus during the Dix–Hallpike or side-lying test for posterior canal BPPV and the supine-roll test for lateral canal BPPV. Video head impulse testing (vHIT) has largely replaced caloric testing worldwide, providing objective vestibulo-ocular reflex (VOR) gain measurement [5]. During vHIT, the examiner delivers brief, passive, high-speed head impulses of 10°–20° amplitude in each canal’s plane to quantify VOR function [6]. This video-based method tests all 6 canals independently; normal VOR gain is ≥ 0.8 in lateral canals and ≥ 0.7 in vertical canals [7].

Fallahnezhad et al. reported reduced posterior-canal VOR gain in unilateral posterior canal BPPV, challenging the assumption of normal VOR in these cases [8]. Aslan et al. observed reduced vertical-canal VOR gains in posterior canal BPPV on vHIT [9]. Karawani et al. showed that vHIT can facilitate rapid BPPV diagnosis, particularly when conventional tests are negative [10]. Tarnutzer et al. identified isolated posterior-canal dysfunction on vHIT in posterior canal BPPV [11]. Guan et al. and Salturk and Yetiser reported decreased VOR gains in posterior canal BPPV compared with healthy controls [12, 13]. Califano et al. found canal dysfunction in 10 of 150 posterior canal BPPV patients, with VOR gain improving after canalith repositioning [14]. A meta-analysis by Elsherif et al. confirmed vHIT’s diagnostic value in posterior canal BPPV, showing significantly reduced VOR gain versus the unaffected side [15].

However, universally accepted abnormal VOR gain thresholds for BPPV remain undefined, and standardized pre- and post-treatment reference values are lacking. This study evaluated vHIT results in BPPV patients before canalith repositioning maneuver (CRM), immediately after treatment, and 1 week post-treatment to assess its diagnostic and monitoring utility.

## Materials and methods

### Study design and period

We conducted a prospective before–after study from April 2022 to July 2023.

### Participants

Inclusion criteria were: (1) age > 18 years; (2) spinning sensation triggered by head-position changes; (3) positive positional or positioning testing; and (4) symptom onset within 4 weeks.

Exclusion criteria were: (1) central vertigo or neurological deficits; (2) psychiatric disorders or attention deficits; (3) head or cervical spine injury or restricted neck mobility; (4) severe hypertension; and (5) blurred vision.

Withdrawal criteria were: (1) inability to tolerate positional testing (Dix–Hallpike, supine-roll, or side-lying tests) or CRMs; and (2) intolerance to vHIT.

### Sample size calculation

Sample size calculation was based on prior data assuming a mean VOR gain of 0.8 with a standard deviation of 0.12 [15]. To achieve a 95% confidence interval with a margin of error of ± 0.03, we required 63 patients with BPPV.

### Ethics, recruitment, and consent

After institutional review board approval (Faculty of Medicine Siriraj Hospital, Si-223/2022), patients with suspected BPPV were invited to participate and provided written informed consent.

### Baseline patient-reported outcomes

Each participant completed the Dizziness Handicap Inventory–Thai version (DHI-T) before treatment to assess self-perceived disability [16].

### Diagnostic positional testing

Initial diagnostic evaluation used the Dix–Hallpike, side-lying, or supine-roll tests. Patients with findings consistent with BPPV were enrolled.

### vHIT protocol and time points

The vestibulo-ocular reflex (VOR) was evaluated using the ICS^®^ Impulse video head impulse testing (vHIT) system (GN Otometrics, Taastrup, Denmark). All assessments followed a standardized clinical protocol [6].:

- Instrumentation and Calibration: The system employed a high-speed infrared camera with a 250 Hz sampling frequency. Before data collection, a standard laser-point calibration was performed at a distance of 1 m, during which the patient was instructed to track alternating targets using only eye movements.
- Data Acquisition Protocol: At least 20 valid head impulses were recorded for each semicircular canals. Impulses were manually delivered by the examiner with a small amplitude (10 °-20°) and high acceleration (1000 °/s²-2500°/s²), targeting a peak head velocity range of 150°/s to 250°/s.
- Artifact Management: Traces were subjected to both automated and manual screening to ensure data integrity. Rejection criteria included goggle slippage (characterized by eye velocity leading head velocity) and palpebral interference (blinks).
- Quantitative Analysis: VOR gain was determined using the Area Under the Curve (AUC) method, calculated as the ratio of the eye velocity area to the head velocity area from the onset of the impulse until head velocity returned to zero.
- Saccadic Evaluation: The presence, latency, and amplitude of covert and overt corrective saccades were systematically analyzed to supplement gain measurements and enhance the detection of vestibular deficits.

vHIT was performed before any therapeutic intervention to quantify VOR gain in all semicircular canals. It was repeated immediately after treatment and again at the 1-week follow-up. Normal VOR gain is ≥ 0.8 in lateral canals and ≥ 0.7 in vertical canals [7].

### CRMs

All participants received CRMs specific to the affected canal: Epley or Semont maneuver for posterior and anterior canal BPPV, and Barbecue or Gufoni maneuver for lateral canal BPPV.

### Follow-up and outcomes

**Fig. 1:**
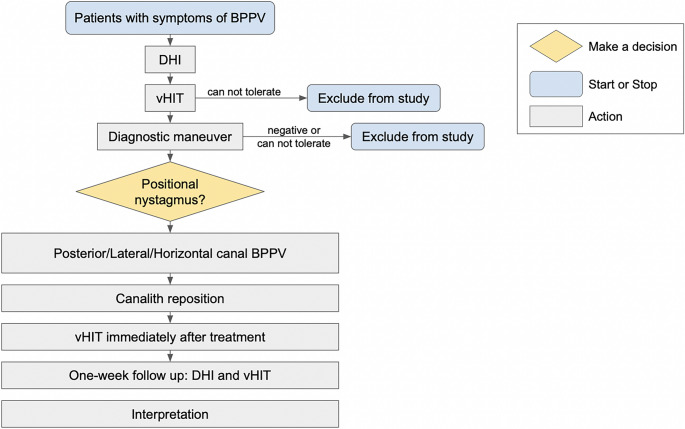
Study flow diagram showing participant screening, enrollment, canalith repositioning, and scheduled assessments. vHIT was obtained pretreatment, immediately after the canalith repositioning maneuver, and at 1-week follow-up. The DHI-T was completed at baseline and at 1 week. Abbreviations: BPPV, benign paroxysmal positional vertigo; CRM, canalith repositioning maneuver; DHI-T, Dizziness Handicap Inventory–Thai version; SCC, semicircular canal; vHIT, video head impulse test; VOR, vestibulo-ocular reflex. (Created in Microsoft Word)

Participants returned at 1 week for repeat vHIT and DHI-T. VOR gain values and DHI-T scores from pre-treatment, immediate post-treatment, and 1-week follow-up were recorded for analysis. The flow of the study is illustrated in Fig. 1.

### Outcomes

The primary outcome was VOR gain measured by vHIT in the affected semicircular canal at 3 time points: pre-treatment, immediate post-CRM, and 1 week post-treatment. The secondary outcome was the change in DHI-T scores, assessed before treatment and at 1-week follow-up.

### Statistical analysis

We used descriptive statistics to summarize baseline characteristics and outcomes. Unpaired *t* tests were applied to compare pre-treatment VOR gain across categorical variables (sex, diabetes mellitus, hypertension).

To evaluate the impact of clinical variables—including the affected canal, symptom duration, and prior vestibular suppressant use—on changes in VOR gain over time (pre-treatment, immediately post-treatment, and one-week follow-up), a repeated-measures analysis of variance (rm-ANOVA) was conducted on complete cases. The assumption of sphericity was assessed; in instances where this assumption was violated (*p* < 0.05), multivariate tests were utilized. Effect sizes were quantified using partial eta-squared, with values < 0.06 categorized as small effects.

To account for the 12% missing data rate post-treatment, a sensitivity analysis was performed using a Generalized Estimating Equation (GEE) (Supplemental Table 1) with an exchangeable correlation structure. In this model, baseline (pre-treatment) VOR gain was included as the primary covariate; additional clinical factors were excluded as they did not significantly influence VOR gain in preliminary analyses. Finally, changes in DHI-T scores from baseline to the one-week follow-up were analyzed using rm-ANOVA.

Pearson’s correlation coefficient (*r*) quantified the linear relationship between VOR gain and DHI-T at baseline and 1 week post-treatment. All analyses used IBM SPSS Statistics version 29 (IBM Corp, Armonk, NY, USA).

## Results

### Patient characteristics

Sixty-three patients with BPPV were included. Most were female (*n* = 52, 82.5%); age ranged from 24 to 85 years (mean 59.9 ± 13.1 years). Posterior canal BPPV occurred in 51 patients, lateral canal in 11, and anterior canal in 1. Right ear involvement was observed in 35 patients (55.6%). Nearly half (49.2%) reported vertigo lasting < 1 week, and 36.5% had used vestibular suppressants before the hospital visit (Table 1).

**Table 1:** Baseline characteristics and pre-treatment VOR gain by subgroup in BPPV

Characteristics	Number (%)	VOR gain: Pretreatment	Mean difference	*p* Value
		(Mean ± SD)	(95% CI)	
Underlying disease, Yes	50 (79.4)			
Diabetes mellitus	12 (19)			
Hypertension	24 (38.1)			
Dyslipidemia	26 (41.3)			
Affected canal				
Posterior	51 (81.0)			
Lateral	11 (17.4)			
Anterior	1 (1.6)			
Affected side				
Right	35 (55.6)			
Left	28 (44.4)			
Onset of vertigo (wk)				
< 1	31 (49.2)			
≥ 1	32 (50.8)			
Prior vestibular suppressant medication				
Yes	23 (36.5)			
No	40 (63.5)			
Age (y)	63	59.9 ± 13.1	–	–
Sex				
Male	10 (15.9)	1.001 ± 0.154		
Female	53 (84.1)	1.002 ± 0.176	0.001 (− 0.118, 0.121)	0.983
DM				
No	51 (81.0)	0.985 ± 0.177		
Yes	12 (19.0)	1.073 ± 0.129	0.088 (− 0.021, 0.197)	0.110
Hypertension				
No	39 (61.9)	0.991 ± 0.157		
Yes	24 (38.1)	1.020 ± 0.196	0.028 (− 0.061, 0.118)	0.529
Dyslipidemia				
No	37 (58.7)	1.005 ± 0.153		
Yes	26 (41.3)	0.998 ± 0.198	−0.007 (− 0.095, 0.082)	0.879
Onset (week), Yes				
< 1	31 (49.2)	1.004 ± 0.160		
≥ 1	32 (50.8)	1.000 ± 0.184	−0.004 (− 0.091, 0.084)	0.935
Prior vestibular suppressant				
No	40 (63.5)	0.985 ± 0.175		
Yes	23 (36.5)	1.032 ± 0.164	0.047 (− 0.043, 0.136)	0.302
Affected canal				
Posterior	51 (81.0)	0.990 ± 0.186		
Lateral	11 (17.5)	1.062 ± 0.071	0.072 (0.004, 0.140)	0.039*
Anterior	1 (1.6)	–		

Values are mean ± SD unless indicated. Mean difference and 95% CI compare each subgroup with the reference. Normal VOR gain: lateral ≥ 0.8; vertical ≥ 0.7

* *p* < 0.05 denotes significance

Abbreviations: *BPPV* benign paroxysmal positional vertigo, *CI* confidence interval, *DM* diabetes mellitus, *SD* standard deviation, *VOR* vestibulo-ocular reflex, *wk* week, *y* year

### Follow-up and attrition

The initial cohort included 63 patients. One patient was excluded for failing to undergo a vHIT immediately following treatment, leaving 62 patients for the initial assessment. At the 1-week follow-up, 8 patients were lost to follow-up, leaving 55 patients who completed the vHIT. Of these, 2 patients did not complete the DHI-T questionnaire, resulting in 53 sets of patient-reported outcomes. Longitudinal analyses were restricted to complete cases only (*N* = 54, consisting of 43 posterior and 11 lateral canal cases), excluding the single anterior canal case to maintain statistical homogeneity and power. Sensitivity analysis was performed to evaluate the impact of these missing cases, confirming that they did not differ demographically from the analyzed cohort.

### vHIT findings

#### Pre-treatment assessment (*N* = 63)

Baseline comparisons of VOR gain are presented in Table 2. VOR gain did not differ by sex, underlying diseases, onset of vertigo, or prior vestibular suppressant use. Mean VOR gain in the affected posterior canal was significantly lower than in the lateral canal (0.990 vs. 1.062; *p* = 0.039). Lateral canal gain values slightly exceeding 1.0 may be attributable to various technical artifacts. These include calibration errors, head overshoot, and anticipatory eye movements, as well as goggle slippage caused by an insufficiently secured strap; these allow the eye velocity tracing to lead the head velocity, resulting in an artificially elevated VOR gain. Abnormal VOR gain (≤ 0.7) occurred in 4 right posterior canal cases (6.35%), all females. All lateral canal BPPV cases had normal VOR gain. No overt or covert saccades were observed.

**Table 2:** VOR gain across treatment time points by affected canal

		Posterior canal		Lateral canal	
	*N*	*n*	Mean ± SD	*n*	Mean ± SD
Pretreatment	63	51	0.990 ± 0.186	11	1.062 ± 0.071
Immediate posttreatment	62	50	1.002 ± 0.206	11	1.057 ± 0.107
One-week posttreatment	55	44	0.966 ± 0.179	11	1.049 ± 0.117

Values are mean ± SD. Time points: pretreatment, immediate post-CRM, and 1 week. Sample sizes: pretreatment *N* = 63, immediate posttreatment *N* = 62, 1 week *N* = 55. Normal VOR thresholds as above

Abbreviations: *CRM* canalith repositioning maneuver, *SD* standard deviation, *VOR* vestibulo-ocular reflex, *n* sample size, *N* total sample

### Immediate post-treatment assessment (*N* = 62)

One patient did not undergo vHIT immediately after treatment. Among the remaining 62 patients, mean VOR gain was 1.002 in the treated posterior canal and 1.057 in the lateral canal (Table 2). Abnormal gain (≤ 0.7) was seen in 3 patients, all in the right posterior canal; 1 had shown reduced VOR gain during the pre-treatment assessment.

#### One-week assessment (*N* = 55)

Fifty-five patients completed the 1-week assessment. Almost all reported no residual dizziness. The mean VOR gain was 0.966 in the treated posterior canal and 1.049 in the lateral canal (Table 2). Three patients had low gain, all in posterior canals. Of these 3 patients with low gain at 1 week, only 1 had shown abnormal baseline VOR gain (≤ 0.7); this value improved but remained abnormal. Among the 4 patients with abnormal baseline VOR gain, 3 normalized after treatment.

#### Summary across time points

Table 2 shows VOR gain by affected canal at all 3 time points; Table 3 compares gains across time points using rm-ANOVA based on 55 patients with complete data. VOR gain showed no significant change across time points and no differences by affected canal, onset of vertigo, or prior vestibular suppressant use. Due to 12% missing VOR gain after treatment, sensitivity analysis of VOR gain performed using GEE (Supplemental Table 1) on all patients reveal findings consistent with those obtained from the rm-ANOVA.

**Table 3:** Repeated-measures (rm) ANOVA of VOR gain over time by clinical factors

Group		VOR: Mean ± SD			*p* Value*		
	*n*	Pretreatment	Immediate posttreatment	1 week posttreatment	Group	Time	GroupxTime
Canal							
Posterior	43	0.993 ± 0.193	0.992 ± 0.201	0.968 ± 0.181	0.156	0.780	0.958
Lateral	11	1.062 ± 0.071	1.057 ± 0.107	1.049 ± 0.117			
Anterior	1	–	–	–			
Lateral – Posterior: Mean diff (95% CI)		0.069 (-0.051, 0.189)	0.065 (-0.062, 0.192)	0.081 (-0.034, 0.197)			
Partial Eta-Squared		0.025	0.020	0.037			
Onset of vertigo							
< 1 wk	26	0.999 ± 0.164	0.992 ± 0.143	1.003 ± 0.128	0.919	0.797	0.596
≥ 1 wk	29	1.012 ± 0.188	1.018 ± 0.218	0.976 ± 0.209			
≥ 1 wk - < 1 wk: Mean diff (95% CI)		0.014 (-0.082, 0.110)	0.026 (-0.075, 0.127)	-0.027 (-0.122, 0.068)			
Partial Eta-Squared		0.002	0.005	0.006			
Prior vestibular suppressant							
No	35	0.985 ± 0.176	0.975 ± 0.154	0.986 ± 0.157	0.231	0.481	0.275
Yes	20	1.043 ± 0.173	1.059 ± 0.225	0.994 ± 0.205			
Yes – No: Mean diff (95% CI)		0.058 (-0.040, 0.157)	0.084 (-0.018, 0.187)	0.008 (-0.091, 0.107)			
Partial Eta-Squared		0.026	0.049	0.0005			
Total	55	1.006 ± 0.176	1.005 ± 0.185	0.989 ± 0.174	–	0.714	–

* One-way rm-ANOVA was based on 55 complete cases. Effects shown for group, time, and group-by-time interaction. Values are mean ± SD unless indicated

Abbreviations: *ANOVA* analysis of variance, *n* sample size, *SD* standard deviation, *VOR* vestibulo-ocular reflex, *wk* week

#### DHI-T

Fifty-three patients completed both pre- and post-treatment DHI-T assessments (*N* = 53). The baseline mean (SD) DHI score was 32.0 (± 26.2), indicating moderate quality-of-life impact. DHI scores improved significantly from baseline to 1 week (Table 4). However, improvement did not differ by affected canal, onset of vertigo, or prior vestibular suppressant use (Table 4).

**Table 4:** Change in DHI-T from baseline to 1 week by clinical factors (rm-ANOVA)

		DHI-T: Mean ± SD		Mean diff	*p* Value*		
	*n*	Pretreatment	1 week posttreatment	(95% CI)	Group	Time	GroupxTime
Canal							
Posterior	42	46.6 ± 21.4	31.6 ± 26.7	15.0 (7.9, 22.0)	0.487	< 0.001	0.411
Lateral	10	55.2 ± 23.5	33.6 ± 26.5	21.6 (7.1, 36.1)			
Anterior	1	–	–	–			
Onset of vertigo							
< 1 week	25	49.5 ± 18.6	33.3 ± 23.2	16.2 (7.0, 25.5)	0.737	< 0.001	0.887
≥ 1 week	28	47.9 ± 24.9	30.8 ± 29.0	17.1 (8.4, 25.9)			
Prior vestibular suppressant							
No	33	45.9 ± 21.9	29.0 ± 26.0	17.0 (8.9, 25.0)	0.211	< 0.001	0.919
Yes	20	53.2 ± 21.7	36.9 ± 26.5	16.3 (6.0, 26.6)			
Total	53	48.7 ± 21.9	32.0 ± 26.2	16.7 (10.4, 23.0)	–	< 0.001	–

One-way repeated-measures ANOVA. “Mean diff (95% CI)” is baseline − 1 week; positive differences indicate improvement. Effects shown for group, time, and group-by-time interaction

* *p* < 0.001 for time indicates significant improvement

Abbreviations: *CI* confidence interval, *DHI-T* Dizziness Handicap Inventory–Thai version, *n* sample size

#### Correlation between VOR gain and DHI-T

**Fig. 2:**
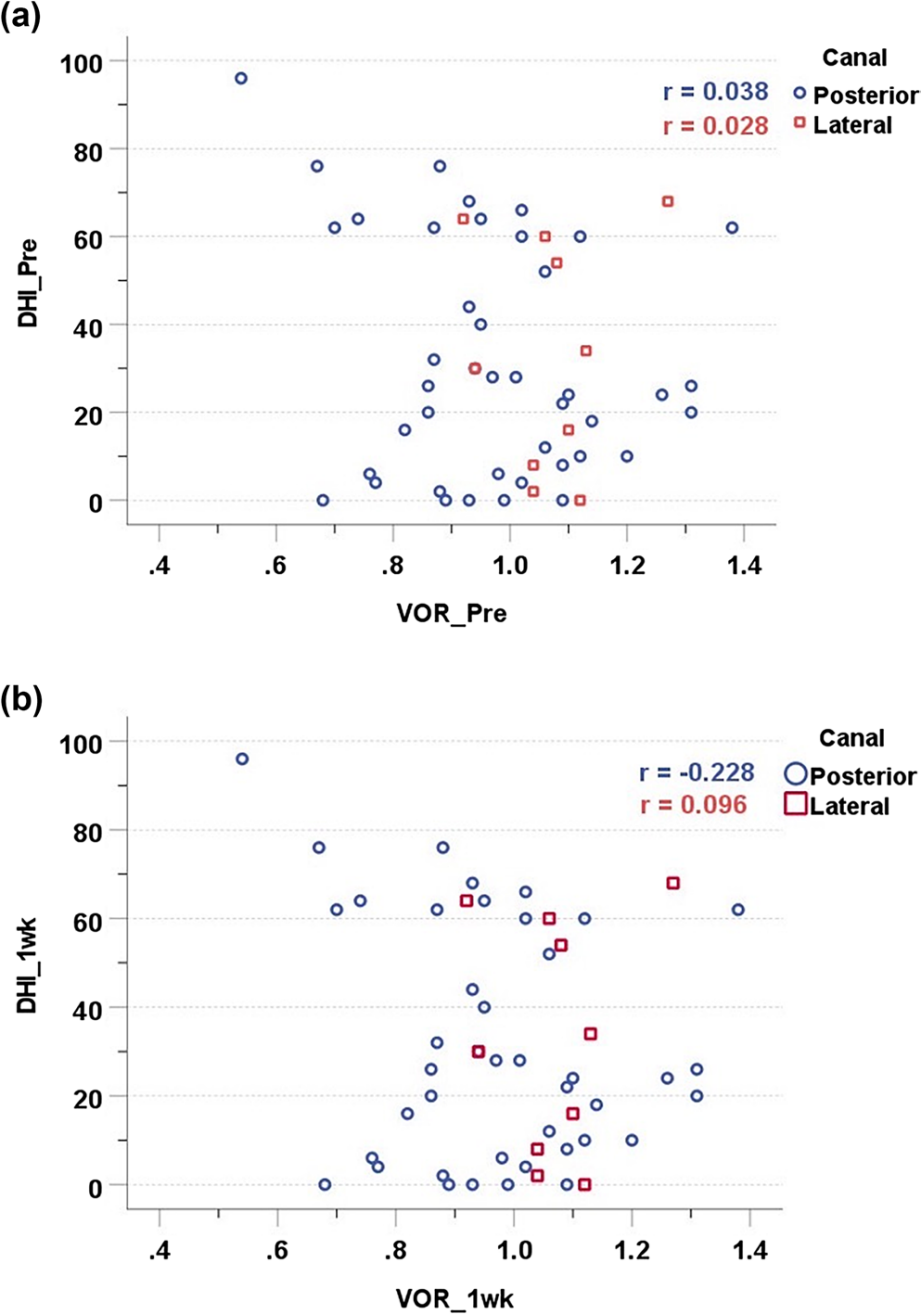
Pearson correlation between DHI-T scores and mean VOR gain at baseline and at 1-week follow-up. Panel (**a**) shows pretreatment values and panel (b) shows 1-week posttreatment values. Normal VOR gain is ≥ 0.8 in lateral semicircular canals and ≥ 0.7 in vertical semicircular canals. Pearson correlation (r) was used to evaluate the linear association between DHI-T and VOR gain. (**b**) 1-week posttreatment. Abbreviations: BPPV, benign paroxysmal positional vertigo; DHI-T, Dizziness Handicap Inventory–Thai version; SCC, semicircular canal; vHIT, video head impulse test; VOR, vestibulo-ocular reflex. Created using SPSS

For pre-treatment, there was no correlation existed between VOR gain and DHI-T scores for either affected canal (*p* = 0.809 and 0.939; Fig. 2a). At 1 week, a weak negative correlation was observed for the posterior canal (*r* = − 0.228; *p* = 0.146) but not for the lateral canal (*r* = 0.096; *p* = 0.793; Fig. 2b).

#### Cases with abnormal pre-treatment VOR gain

Four patients—all female with posterior canal BPPV—had abnormal pre-treatment VOR gain (≤ 0.7) in the affected right posterior canal. Their ages ranged from 63 to 77 years; only 1 had used vestibular suppressants before presentation. VOR gain normalized by 1 week in 3 cases; in the fourth case, gain improved but remained abnormal. Additional data are provided in Table 5.

**Table 5:** Clinical features and VOR/DHI-T trajectories in 4 cases with abnormal pre-treatment VOR

Case	Age, y	Sex	Underlying disease	Affected canal	Side	Onset of vertigo	Prior vestibular suppressant	VOR gain pretreatment	VOR gain immediately posttreatment	VOR gain 1-wk posttreatment	DHI-T pretreatment	DHI-T 1-wk posttreatment
1	77	Female	Multiple myeloma, hypertension, dyslipidemia	Posterior	Right	≥ 1 wk	None	0.66	0.77	0.74	52	64
2	63	Female	Right keratosis obturans	Posterior	Right	≥ 1 wk	Betahistine 24 mg	0.61	0.81	0.7	56	62
3	73	Female	Left cholesteatoma	Posterior	Right	< 1 wk	None	0.56	0.9	0.67	72	76
4	77	Female	Chronic rhinitis	Posterior	Right	≥ 1 wk	None	0.69	0.65	0.84	–	–

Abnormal vertical-canal VOR gain defined as ≤ 0.7. All 4 cases involved the right posterior canal. DHI-T values are total scores. Medication names and doses reflect presenting therapy

Abbreviations: *DHI-T* Dizziness Handicap Inventory–Thai version, *VOR* vestibulo-ocular reflex, *wk* week, y years

## Discussion

### Clinical background

BPPV is the most common peripheral vestibular disorder, producing brief recurrent positional vertigo—typically provoked by lying down or turning while supine—lasting less than 1 min. The pathophysiology involves canalolithiasis or cupulolithiasis in any semicircular canal. Most cases localize to a single canal, most often the posterior canal; however, concomitant posterior and lateral canal involvement in the same ear can occur. Although diagnostic maneuvers are routine, nystagmus may have small amplitude, making it inconspicuous or difficult to detect.

### Role of vHIT

vHIT objectively records head and eye velocities to quantify VOR and assess individual semicircular canal function. This test uses high-frequency passive head rotations as physiological stimuli for canal-specific assessment.

### Canal involvement and abnormal VOR gain

We aimed to describe the time course of vHIT parameters in BPPV before treatment, immediately after treatment, and 1 week post-treatment. Canal involvement was posterior in 51 patients (81%), lateral in 11 (17.4%), and anterior in 1 (1.6%). Before treatment, abnormal VOR gain occurred in 4 of 63 patients (6.35%). This rate aligns with prior reports: Cinar et al. found abnormal posterior-canal VOR gain in 2 of 24 patients (8.3%), and Califano et al. in 10 of 150 (6.67%) [14, 17].

### Age, sex, comorbidity, and ear pathology

All 4 abnormal cases exceeded our cohort’s mean and median ages (59.9 and 54.5 years, respectively). This suggests greater vHIT sensitivity to posterior-canal dysfunction in older individuals and possible age-related VOR gain decline. All 4 were female, consistent with evidence that increasing age and female sex are common BPPV risk factors [18]. Diabetes mellitus, hypertension, dyslipidemia, symptom onset, and prior vestibular suppressant use did not influence vHIT results. Nevertheless, several conditions—vestibular neuronitis, labyrinthitis, head trauma, migraine, inner ear surgery, and Meniere’s disease—are recognized risk factors for BPPV [18]. Two of the four abnormal cases presented with preexisting ear pathologies, specifically cholesteatoma and keratosis obturans, suggests that these comorbidities may have predisposed the patients to BPPV, thereby influencing the study results. However, these patients were included as they strictly met the diagnostic criteria for positional nystagmus during maneuvers.

### VOR gain in typical BPPV

In typical BPPV, VOR gain in the posterior and lateral canals appears largely preserved. However, canal-specific analyses revealed subtle differences between posterior (*n* = 51) and lateral (*n* = 11) canal groups. At baseline, mean VOR gain in the affected posterior canal was significantly lower than in the lateral canal (0.990 vs. 1.062; *p* = 0.039), though both remained within normal ranges. These findings imply subtle canal-dependent differences rather than global vestibular hypofunction.

### Stability across treatment

Across time, VOR gain did not differ statistically before CRM, immediately after treatment, or at 1 week. This aligns with Cinar et al. (*n* = 24) and Califano et al. (*n* = 150), who also found no pre–post treatment differences [14, 17].

### Mechanistic and methodological considerations

The complete absence of corrective saccades in our cohort is a significant finding. It emphasizes that BPPV is fundamentally a mechanical overstimulation of the canal by otoconia rather than a paretic vestibular lesion that would result in a saccadic refixation, which may explain why vHIT parameters remain stable. However, it must be acknowledged that subtle vestibular deficits may fall below the detection threshold of vHIT and cannot be entirely excluded. Accurate vHIT requires an experienced examiner delivering precise, rapid head impulses; reliability improves when a single operator performs all tests. Furthermore, several studies consider vHIT abnormal only when vestibular function decreases by more than 40% [17].

### Patient-reported outcomes vs. vHIT

The mean DHI-T score decreased significantly after treatment (*p* < 0.01). Ideally, higher DHI-T scores would correlate with lower VOR gain, yet we observed discordance. This mismatch likely reflects that most VOR values remained within normal limits, limiting observable correlations. In a few atypical cases, DHI-T scores remained high despite normalized VOR gain. This discordance highlights that vHIT measurements do not always capture the subjective disability of vertigo, particularly when physiological VOR remains within or returns to normal limits.

The mean DHI-T score decreased significantly after treatment (*p* < 0.01). Ideally, higher DHI-T scores would correlate with lower VOR gain, yet we observed discordance. This mismatch likely reflects that most VOR values remained within normal limits, limiting observable correlations. In a few atypical cases, DHI-T scores remained high despite normalized VOR gain. This discordance suggests that vHIT measurements may not fully capture the subjective disability of vertigo, particularly when physiological VOR remains within or returns to normal limits. Furthermore, persistent or worsened DHI-T scores may be influenced by factors unrelated to BPPV, including underlying psychological components, such as anxiety, or the early manifestation of chronic vestibular syndromes, particularly persistent postural-perceptual dizziness (PPPD). The outcomes of the four abnormal cases demonstrated several clinical discordances:

- Case 1: Despite a negative Dix-Hallpike maneuver at the one-week follow-up, the patient’s persistence of symptoms suggest that residual otoconial debris may have remained sufficient to provoke subjective vertigo without triggering a nystagmus response. Additionally, age-related deterioration in central compensation may contribute to prolonged symptom perception. Therefore, the DHI-T score may reflect a clinical worsening despite objective improvement or normalization of VOR gain.
- Case 2: The patient reported symptomatic improvement with only mild residual dizziness and a negative Dix-Hallpike maneuver. However, the high DHI-T score at the second evaluation may be attributed to recall bias or an overestimation of symptom severity compared to the baseline assessment.
- Case 3: Case findings were consistent across all diagnostic modalities; the patient remained symptomatic with a positive Dix-Hallpike maneuver and a correspondingly elevated DHI-T score at the one-week follow-up. These subjective outcomes correlated with vHIT results that, although showing partial improvement, remained within the abnormal range, necessitating a repeat CRM. As with the prior case, advanced age may further impede central compensatory mechanisms, contributing to slower clinical recovery.

### Clinical implications and conclusion

For the diagnosis of BPPV, a detailed clinical history and the performance of provocative positional maneuvers remain the gold standard and essential diagnostic tools. While the vHIT is an increasingly valuable tool in general vestibular assessment, evidence supporting its utility in the routine management of BPPV remains limited. Both current literature and our findings suggest that vHIT may have limited incremental value in the routine evaluation of isolated BPPV cases.

Several limitations of the present study should be considered. The relatively short follow-up period of one week may be insufficient to capture long-term physiological changes in the vestibulo-ocular reflex (VOR). Furthermore, the predominance of posterior canal BPPV cases in our cohort may have biased the results toward a uniform direction, and the exclusion of patients with central or mixed vestibular disorders limits the generalizability of these findings. Consequently, further large-scale, prospective studies with extended follow-up periods are warranted to more definitively establish the role of vHIT in varied BPPV presentations.

## Supplementary Information

Below is the link to the electronic supplementary material.

Supplementary Material 1 (DOCX 14.8 KB)
