# Supplementary material for: Video head impulse testing before and after canalith repositioning for benign paroxysmal positional vertigo
Source: Eur Arch Otorhinolaryngol. 2026 May 5;283(8):5037–46. doi: 10.1007/s00405-026-10263-3 (PMC13407729; doi:10.1007/s00405-026-10263-3)
Supplement: Supplementary file 1 — Supplementary Material 1 (DOCX 14.8 KB) [file 405_2026_10263_MOESM1_ESM.docx]

**Supplemental Table 1 GEE (with exchangeable correlation) of VOR gain after treatment (immediate posttreatment, 1 week posttreatment)**

|  | p-value | | | |
| --- | --- | --- | --- | --- |
| Group | Group | Time | Group-Time interaction | Pre-treatment VOR gain |
| Canal (Posterior, Lateral) | 0.358 | 0.416 | 0.606 | <0.001 |
| Onset of vertigo (< 1, ≥ 1 week) | 0.375 | 0.362 | 0.578 | <0.001 |
| Prior vestibular suppressant (No, Yes) | 0.977 | 0.267 | 0.342 | <0.001 |

Effects shown for group, time, and group-by-time interaction.
*Abbreviations:* VOR, vestibulo-ocular reflex
